# Evaluating the Quality of Serial EM Sections with Deep Learning

**DOI:** 10.1093/mam/ozae033

**Published:** 2024-05-03

**Authors:** Mahsa Bank Tavakoli, Josh L Morgan

**Affiliations:** Department of Ophthalmology and Visual Sciences, Washington University in St. Louis, Euclid Ave., St. Louis, MO 63110, USA; Department of Neuroscience, Washington University in St. Louis, 660 S. Euclid Ave., St. Louis, MO 63110, USA; Department of Biomedical Engineering, Washington University in St. Louis, 1 Brookings Drive, St. Louis, MO 63130, USA; Department of Ophthalmology and Visual Sciences, Washington University in St. Louis, Euclid Ave., St. Louis, MO 63110, USA; Department of Neuroscience, Washington University in St. Louis, 660 S. Euclid Ave., St. Louis, MO 63110, USA; Department of Biomedical Engineering, Washington University in St. Louis, 1 Brookings Drive, St. Louis, MO 63130, USA

**Keywords:** convolutional neural networks (CNNs), deep learning, image quality evaluation, serial section scanning electron microscopy (ssSEM)

## Abstract

Automated image acquisition can significantly improve the throughput of serial section scanning electron microscopy (ssSEM). However, image quality can vary from image to image depending on autofocusing and beam stigmation. Automatically evaluating the quality of images is, therefore, important for efficiently generating high-quality serial section scanning electron microscopy (ssSEM) datasets. We tested several convolutional neural networks for their ability to reproduce user-generated evaluations of ssSEM image quality. We found that a modification of ResNet-50 that we term quality evaluation Network (QEN) reliably predicts user-generated quality scores. Running QEN in parallel to ssSEM image acquisition therefore allows users to quickly identify imaging problems and flag images for retaking. We have publicly shared the Python code for evaluating images with QEN, the code for training QEN, and the training dataset.

## Graphical Abstract

**Graphical Abstract:**
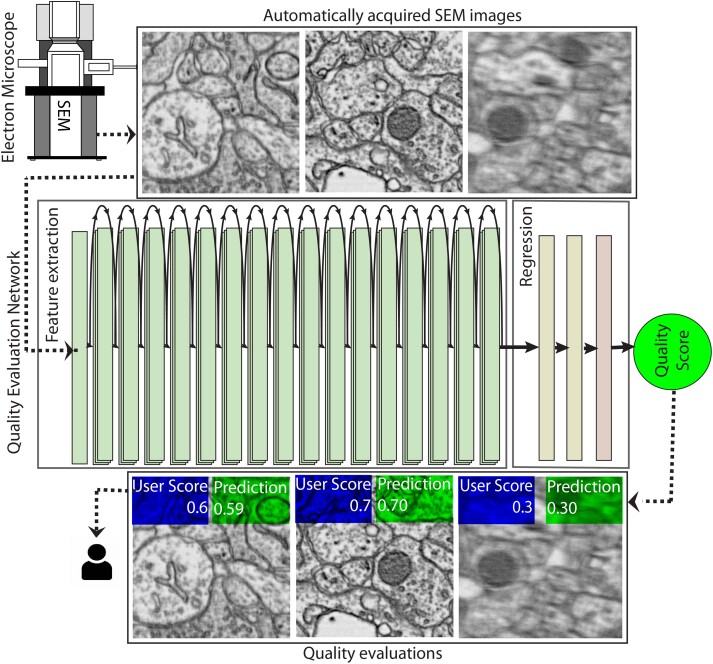

## Introduction

Serial section scanning electron microscopy (ssSEM) can generate high-resolution three-dimensional reconstructions of biological tissue. In this technique, tissue is cut into ultrathin sections (10–90 nm thick), sections are imaged with the scanning electron microscopy (SEM), and images are reconstructed into a three-dimensional digital map of cellular ultrastructure. However, combining high-resolution imaging (∼4--10 nm pixel size) with large tissue volumes (>10μm field of view) can be time-consuming and labor-intensive.

Automating image acquisition, so that SEMs can run 24 h a day without human intervention, has made it easier to apply ssSEM to reconstruct increasingly large image volumes (Hayworth et al., 2014). One of the principal challenges of automating ssSEM is consistently acquiring high-quality images. Good image quality requires that each section is in focus and that the column stigmation is adjusted to account for electrical fields that distort the beam. Image quality can also be affected by the charging of the sample or tissue artifacts. Automatically monitoring the quality of images being produced by the microscope, is, therefore, an important part of maximizing the productivity of an automated ssSEM image acquisition pipeline.

We use the Matlab custom software package WaferMapper (Hayworth et al., 2014) to direct the automatic image acquisition of ultrathin section series. To reconstruct a piece of tissue, hundreds of ultrathin sections are collected on silicon wafers. WaferMapper directs the imaging of the sections at multiple resolutions so that positions of interest within the tissue can be targeted for high-resolution imaging. For each high-resolution image, WaferMapper must drive to the correct position on the wafer, find the correct focus and stigmation, and acquire the image. Built into WaferMapper is the ability to evaluate the quality of the images it acquires with a function called checkQual. This quality value can be used to automatically refocus the image, automatically retake an image, or can serve as a flag for the user to direct manual retaking of the image.

The checkQual function of WaferMapper divides images into 3×3 pixel kernels and measures the contrast within these kernels using different groupings of the kernels. A kernel where more contrast is found by grouping kernels into lines as opposed to speckle patterns is considered to have a high-quality value. The overall image quality is assigned according to the quality value of the best kernels within the image. This approach has the advantage of being fast and robust to large regions of empty space in an image. However, its reliability is limited, and it is very sensitive to signal-to-noise ratio. Because noise increases the unstructured contrast relative to structured contrast, differences in staining intensity, beam intensity, and detection efficiency alter the quality value even when the image focus has not changed.

To find a more robust method of evaluating image quality, we turned to CNNs. In recent years, deep learning techniques have shown remarkable success in improving the performance and accuracy of image processing tasks. Each layer of a CNN encodes increasingly derived image features; starting with pixel values and combining these to recognize increasingly higher-order image features. This hierarchical feature extraction can include recognizing image features corresponding to image degradation (Hou et al., 2015; Yang et al., 2019).

CNNs have been successful in evaluating image quality of natural images (Hou et al., 2015; Sun et al., 2016; Cheng et al., 2017; Gao et al., 2018; Kim et al., 2018). However, there are relatively few blind image quality assessment approaches for medical images. The purpose of medical image quality assessment differs significantly from natural image quality assessment as it assesses images based on their impacts on accomplishing specific tasks (such as the accuracy of diagnoses or the precise localization of anatomical/biological structures) rather than focusing on the general quality of natural images (Ding, 2018). In Zhang et al. (2021), a CNN named DCNN-IQA-14 was developed specifically for evaluating the quality of ultrasound images. The authors found that, while increasing the number of convolution layers did not improve the final peak performance of the network, more layers significantly decreased the number of training epochs required to reach peak performance. For their analysis of ultrasound images, Zhang et al. (2021) found that increasing the number of layers from the 8 convolution layers used in DCNN-IQA-8 (Kim et al., 2019) to 14 convolution layers produced a good trade-off between the number of epochs required for training and the computational time of each training epoch.

Adding more layers does not necessarily improve performance. The experience of He & Sun (2015) and Srivastava et al. (2015) was that performance decreased as layers were added (network degradation problem, He et al., 2016). ResNet, a CNN introduced in He et al. (2016), dealt with this issue by creating shortcut connections in which the output of the current block is added to the output of the previous block. This approach mitigates the network degradation problem and promotes better training of deeper networks. This type of network, CNN with residual blocks and shortcuts, has also been applied specifically to the problem of evaluating the quality of biomedical images (MIQA, Cui et al., 2020).

Here, we trained three previously published CNNs (ResNet-50 He et al., 2016, MIQA Cui et al., 2020, and DCNN-IQA-14 Zhang et al., 2021) to evaluate the quality of ssSEM images. We found that a modified ResNet-50 CNN that we refer to as QEN performed remarkably well in predicting human-assigned quality values.

## Materials and Methods

### Data Acquisition

All animal experiments were performed with Washington University in St. Louis with IACUC approval (22–0151). The images were acquired from a single healthy postnatal day 11 wild-type C57blk6 mouse. Images of the dorsal lateral geniculate nucleus were acquired as part of a study of visual circuit development.

Three hundred micrometer thick mouse brain slices were prepared for electron microscopy (EM) imaging as described previously (double layer osmium, uranyl acetate, and lead aspartate Friedrichsen et al., 2022). A section series of 1,500 40 nm thick sections was collected on conductive tape and silicon wafers for imaging with SEM (Schalek et al., 2012). WaferMapper acquisition software (Hayworth et al., 2014) was used to acquire a 4 nm pixel-size dataset (3.7 trillion voxels total) for circuit reconstruction. We collected 2,500 patches (512×512 pixels) from the full dataset to generate our quality evaluation samples (QES) dataset. Patches were selected to include a range of image qualities. This training data are available here.

### Dataset Preparation

**Fig. 1.**
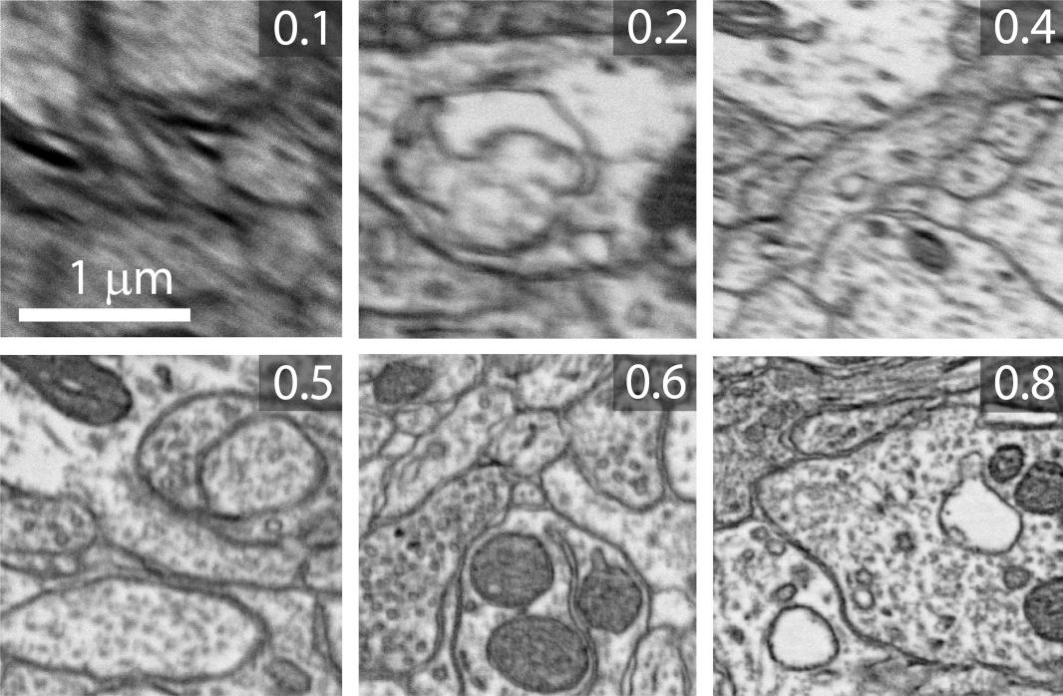
Examples of EM images with subjectively assigned quality scores. Image resolution 4 nm.

For each patch in QES, we manually assigned a quality score from 0.1 to 1 with 1 being the best quality (Fig. 1). These scores were based on the visibility and clarity of biological features such as cell membranes and vesicles. Factors that decrease image quality include poor focus, astigmation, high noise, and tissue artifacts. Two thousand of the samples were used to train the quality evaluation networks while 500 patches were set aside for testing.

### The Architecture of QEN

**Fig. 2.**
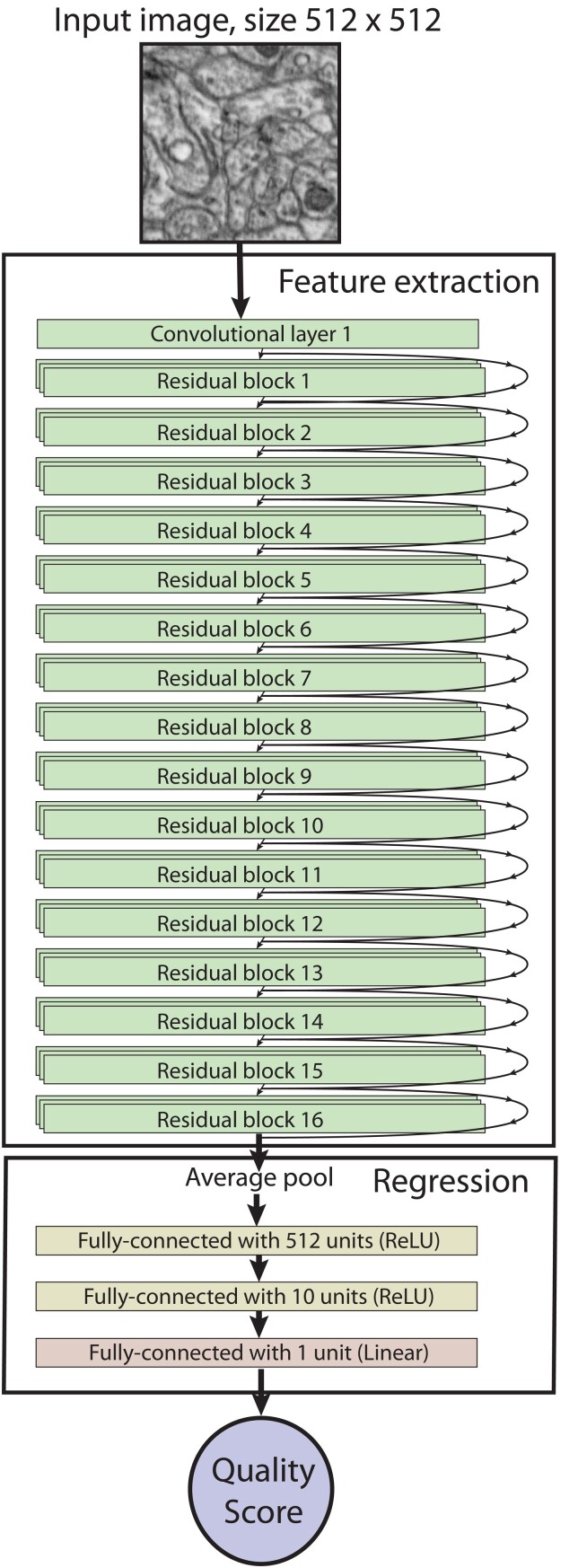
The architecture of QEN. Images are fed into the feature extraction stage composed of an initial convolution layer and 16 residual blocks of 3 layers each. Arrows indicate that outputs are both fed to the subsequent block and added to the output of that block (skip connection). The output of the feature extract stage is fed to a regression stage that generates the quality score.

Our QEN contains two blocks: a feature extraction block and a regression block (Fig. 2). The feature extraction block consisted of the first 49 layers of the 50-layer ResNet-50. ResNet-50 classifies images by first extracting hierarchical features from input images (He et al., 2016). The network contains an initial convolution layer and 16 residual blocks (He et al., 2016). Each block contains three convolution layers. Within each residual block, a “shortcut” connection allows, allowing the original input to bypass the convolution layers and directly merge with the block’s output.

The second, regression, block of our QEN is composed of three layers that compute a single quality score based on features extracted from the first block. For comparison, the last layer of ResNet-50 is a fully connected layer with a softmax activation function designed to perform classification. In our QEN, this layer is replaced with a regression block composed of three fully connected layers. The first two layers consist of 512 and 10 units, respectively. These layers employ the rectified linear unit (ReLU) activation function (Hao et al., 2020), which introduces nonlinearity and helps capture nonlinear dependencies within the data. The final fully connected layer is composed of a single unit with a linear activation function that produces the final quality score.

Our QEN is, therefore, a customization of the ResNet-50 architecture that allows us to leverage ResNet’s powerful feature extraction capabilities (feature extraction block) while tailoring the output to EM image quality evaluation (regression block).

### Comparison Networks

To evaluate the performance of the QEN in relation to the recent advancements in blind quality assessment, we compared our QEN to the MIQA (Zhang et al., 2021) and DCNN-IQA-14 (Cui et al., 2020) networks. The MIQA network is composed of fourteen convolution layers, two down-sampling layers, a pooling layer, and two fully connected layers. On the other hand, the DCNN-IQA-14 network consists of a convolution layer, a max pooling layer, two residual blocks, a minimum pooling layer, and two fully connected layers. Both of these networks (Cui et al., 2020; Zhang et al., 2021) demonstrated superior performance compared to previous networks, such as DCNN-IQA-8 (Kim et al., 2019) and a CNN (Kang et al., 2014). For our comparison to QEN, DCNN-IQA-14 and MIQA were tuned as recommended by Zhang et al. (2021) and Cui et al. (2020). The networks were trained with the QES training dataset as described below for QEN. The exception was that we used image sizes of 244×244 instead of 512×512 for DCNN-IQA-14 to conform with its architecture.

### Training QEN

We started by initializing the weights of the feature extraction block with pretrained ImageNet weights for image classification (He et al., 2016). Aside from reducing the number of epochs required for training, initializing the network with pretrained ImageNet weights should reduce overfitting (Zhang et al., 2021). For each epoch of training, the network calculates a quality score for the QES training images and finds the mean square error (MSE) between that score and the human subjective ground truth. We used the Adaptive Moment Estimation (Adam, Kingma & Ba, 2017) optimizer to change the weights of the network. This algorithm uses gradient-based optimization to minimize the MSE loss function. We set the learning rate to 0.0001 and 0.001. The learning rate controls how much to update the model’s weights in response to the estimated error. In each epoch, 5% of the QES training data was set aside for validation. These images were not used to train the network but were instead used to monitor the model’s performance.

We trained QEN in two stages. In the first stage (10 epochs), the layers of the feature extraction block were frozen and the learning rate was set to 0.0001. These settings allowed the rapid update of the weights in the untrained regression block. In the second stage (40 epochs), all layers of the QEN were made trainable and the learning rate was set to 0.001. This stage allowed for the training of both blocks for the specific quality assessment task.

## Results

### Network Performance

**Fig. 3.**
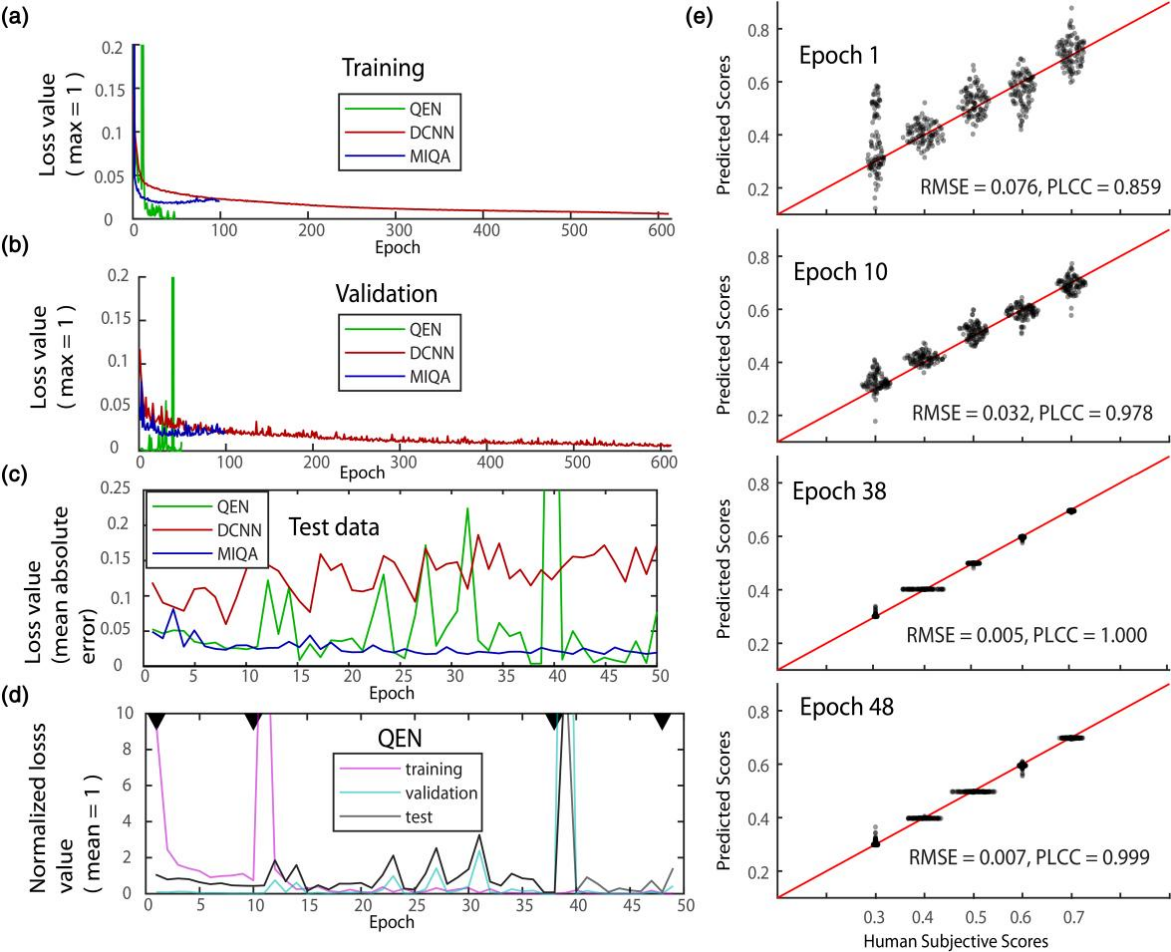
Training networks to evaluate image quality. **(a)** Change in training loss values (max normalized to 1) of three networks over the course of training. **(b)** Loss values of validation data for the same training as **(a)**. **(c)** Mean absolute error for predicting subjective quality scores given to test data. Traces show the performance of three networks over the course of their training. **(d)** Performance of QEN in predicting subjective quality scores. Traces show loss values for training data, loss values for validation data), and mean absolute error for test data). All traces are normalized to mean = 1. Black arrows indicate representative epochs plotted in **(e)**. **(e)** Scatter plots show the relationship between subjectively assigned quality values and the predictions of QEN. Plots for four epochs are shown.

During each training epoch, 95% of the training data was used to update the weights of the network while 5% (validation) was used to independently track the performance of the model. Monitoring the loss values of the three networks during training showed that each network improved significantly within the first 20 epochs (Figs. 3a, 3b). In the subsequent epochs, the performance of QEN became unstable and MIQA bottomed out while DCNN-IQA-14 continued to gradually improve even at our final training of 600 epochs. In later stages of QEN training (epoch 22–50), the validation data loss value increases relative to the training data loss value. This relative increase is indicative of the overfitting of the model. We used this comparison to decide when to terminate the training of each network (Figs. 3c, 3d).

After training, we applied the models generated at each epoch to evaluate the quality of our QES test data. We found that QEN produced the best result with a mean absolute error (MAE) of 0.005, at epoch 38 (Fig. 3e). That compares to a best MAE of 0.017 for MIQA at epoch 31 and an MAE of 0.15 for DCNN-IQA-14 at epoch 207. We therefore focused the rest of our efforts on evaluating image quality with QEN.

In the later stages of QEN training, we saw an instability in performance with several epochs generating very high loss values. We found that the same epochs generated high loss values in both the validation and test data but not the training data (Fig. 3d). This correspondence suggests that the high loss values were not noise in our measurement, but a large difference in the performance of the network due to overfitting.

**Fig. 4.**
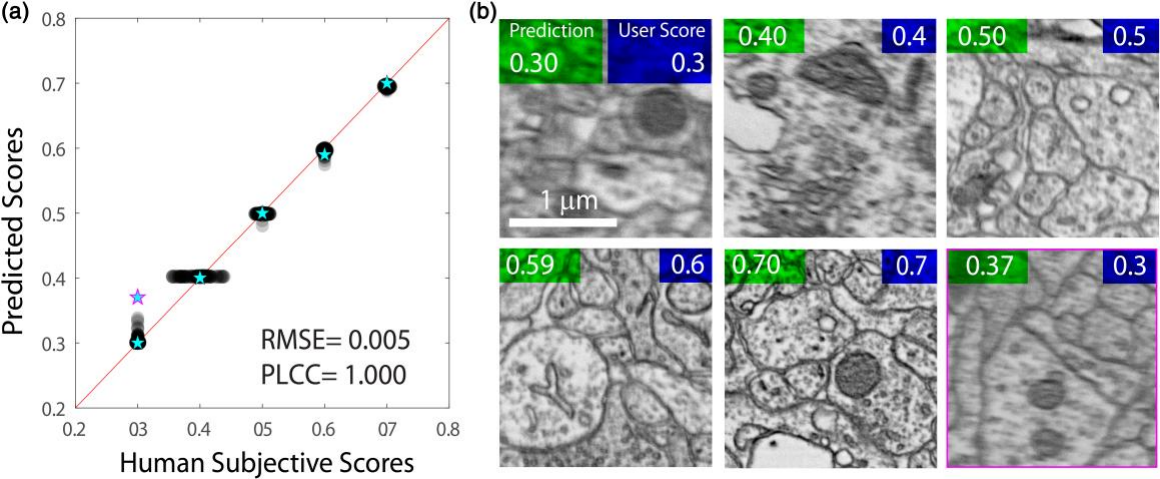
Performance of the best QEN model on the test dataset with examples from outside of the test dataset. **(a)** Scatterplot showing subjective scores of test data relative to predicted scores for the best model (QEN epoch 38). RMSE and PLCC are calculated for the test data. Cyan stars show subjective quality and prediction for six example images from outside the test dataset. Example images are shown in **(b)**. An example of a prediction error outlier is highlighted in magenta (subjective = 0.3 vs predicted = 0.37). **(b)** Examples of EM images from outside the test dataset with QEN assigned score (top left) and human subjective score (top right). An example outlier is highlighted in magenta (blottom right image).

To ensure that model performance increased across all quality values, we plotted subjective ratings of test data against model predictions at various stages of training (Figs. 3d, 3e). We also used the Pearson linear correlation coefficient (PLCC) (Equation 1, Zhang et al., 2021) to measure the extent to which the network was successfully comparing the quality of images independent from the specific values it was using. Among the evaluated models, QEN (Fig. 4) achieved the best error scores (QEN: PLCC = 1.0, root mean square error (RMSE) = 0.005; DCNN-IQA-14: PLCC = 0.59, RMSE = 0.154; MIQA: PLCC = 0.99, RMSE = 0.017). We found that Epoch 38 QEN was reliable, reproducing subjective quality ratings across the range of quality values (Figs. 3e, 4).


(1) $$\mathrm{PLCC}=\frac{\sum_{i=1}^{n}(x_{i}-\bar{x})(y_{i}-\bar{y})}{\sqrt{\sum_{i=1}^{n}(x_{i}-\bar{x}{)}^{2}.\sum_{i=1}^{n}(y_{i}-\bar{y}{)}^{2}}}.$$

### QEN Speed

**Fig. 5.**
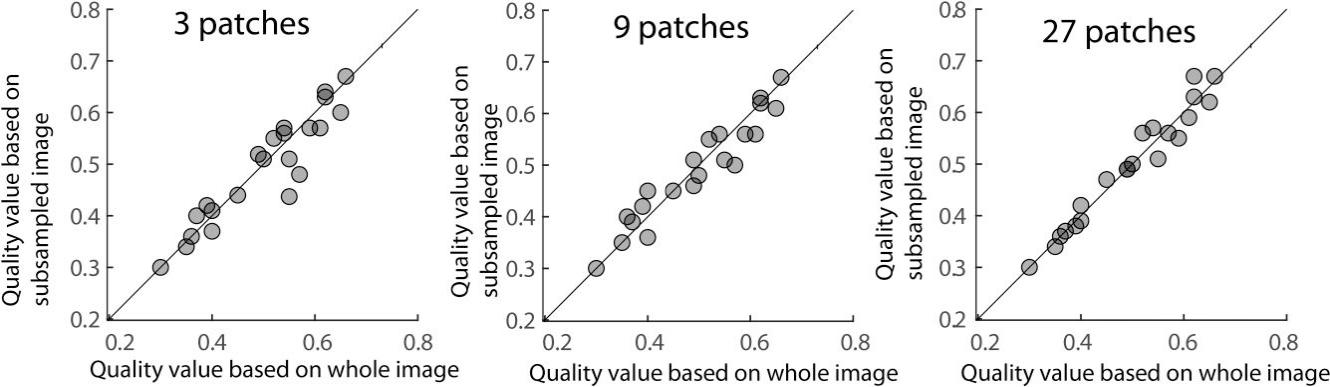
Relationship between the image quality values predicted by evaluating an entire 20,000×20,000 image compared to evaluating 3 (left), 9 (middle), and 27 (right) randomly selected 512×512 patches from the same image.

Evaluating image quality with QEN can be accomplished at rates comparable to image acquisition time using a desktop computer with 8 processors, 4 cores, and 128 G RAM (Intel(R) Xeon(R) W-2123 CPU @ 3.60 GHz). Calculating a quality value for a single 512×512 image patch takes ∼0.2 s. The processing time of ∼0.8μs per pixel is around the speed of our image acquisition (0.1 μs to 1 μs per pixel). A full 20,480×20,480 pixel image, therefore, takes 40–400 s to acquire, 4 s to read, and ∼350 s to analyze the quality. Processing times can be decreased by subsampling the image data. We used the gdal python package to read 512×512 image patches (∼0.3 s read time per patch) from larger EM images. For 21 images, we compared the quality values derived from 1,600 patches for each image (full image) with quality values based on 3, 9, and 27 random patches. We found that the differences in quality values between the fully analyzed image was 0.012 to 0.023 for 3 patches, 0.012 to 0.024 for 9 patches, and 0.007 to 0.016 for 27 patches (95%CI)(Fig. 5). Each image evaluation (reading and analyzing) took around 6 s, 8 s, and 12 s respectively. We were, therefore, able to evaluate image quality reliably in a small fraction of the time it took to acquire the image.

### Availability of QEN

The code for running and training QEN is available at GitHub (here) and can be downloaded and run on Windows or Linux. Running the code requires Python version 3.9 or higher. For postacquisition analysis, the script QEN_evaluate.py can be used to evaluate all images in a folder. For analyzing images as they are acquired, the script QEN_folderMonitor.py will monitor a folder and its subfolders for new images, evaluate them, and update an image quality report spreadsheet. The model we used for our images, QENmodel_epoch38.hdf5, can be accessed from here.

## Discussion

We found that the ability of QEN to recapitulate user-generated scores was good enough that we could trust QEN to monitor our automated image acquisition pipeline. By running the application during acquisition and updating the log of image qualities as new images were acquired, we could document microscope performance, be alerted to imaging problems, and choose which images to retake.

However, for image evaluation to keep up with image acquisition speeds, we only processed subregions of our 20k×20k images. This subsampling did not impact our image evaluation because our image statistics are fairly uniform across the imaging field of view. For other data types, subsampling could miss important clues to image quality. Speeding up image evaluation could be done with faster processors or GPU implementation of image evaluation.

The images we trained and tested QEN on were monochrome 2D images of cell membranes and organelles. The image features that QEN had to detect to evaluate their quality were fairly simple and local: primarily focus, stigmation, and noise. We have not tested how much divergence from this image type can be tolerated before the network needs to be retrained. In particular, other types of EM images and other data types can suffer from higher-order spatial distortions that could require significantly more training to evaluate.

In experimenting with different approaches for evaluating image quality, we tried many different network architectures including VGG, Xception, and Automated Machine Learning using Auto-Keras (Jin et al., 2023). We have provided some data on the networks we tested most extensively, but we do not claim that the differences we observed demonstrate the general performance benefits of one network over others. One of the major features that varied between networks was the number of convolution layers. QEN was a modification of ResNet-50 and had significantly more layers than most other networks tested. It was, therefore, not surprising that this network performed the best. Given the task, available hardware, and pretrained model, the time required to train and execute this 50-layer network was reasonable for our task.

We have made the QES dataset and our manual evaluations of image quality available here so that others can use them to train their networks. We hope that the data provided here provide an opportunity for others to test other approaches to evaluating image quality. In particular, methods that are faster and that can achieve reliable results by reading small areas of images will be particularly helpful for image acquisition.

## Availability of Data and Materials

The code for running and training QEN is available at GitHub https://github.com/MorganLabShare/QEN. Training data is available at download.brainimagelibrary.org/66/62/666264e6585e70ac/. The model we used for our images, QENmodel_epoch38.hdf5, can be accessed from download.brainimagelibrary.org/66/62/666264e6585e70ac/.
